# DOES CHANGE IN INTERNET USE PREDICT PSYCHOLOGICAL WELL-BEING AMONG OLDER ADULTS?

**DOI:** 10.1093/geroni/igz038.1189

**Published:** 2019-11-08

**Authors:** Ronald W Berkowsky

**Affiliations:** California State University Channel Islands, Camarillo, California, United States

## Abstract

Previous work focusing on the relationship between Internet use and quality of life among older adults (aged 65+) has found evidence of various positive impacts. This project expands upon this work by examining the relationship between Internet use and measures of psychological well-being (PWB) including autonomy, environmental mastery, personal growth, positive relations with others, purpose in life, and self-acceptance. The analytic sample is derived from two waves of data (Time 1 = 2004, Time 2 = 2011) taken from the Wisconsin Longitudinal Study and includes a sample of older adults aged**~**65 at Time 1 (N = 4943). Participants were separated into four categories: those who did not use the Internet at Time 1 or 2, those who used the Internet at Time 1 only, those who used the Internet at Time 2 only, and those who used the Internet at both Time 1 and 2. Regression analyses were performed with the Time 2 PWB measures as the outcomes and the Internet use categories as the primary predictors. Results indicate that while continuous Internet users typically reported higher PWB scores compared to non-users, those who stopped use between Time 1 and 2 also reported higher scores and those who started use between Time 1 and 2 reported lower scores. These results generally held when introducing Time 1 PWB measures as controls, suggesting changes in Internet use may affect PWB but not necessarily in the predicted directions. Additional control variables, potential explanations, and implications for future research are discussed.

